# Shared and non-overlapping functions of RECQL4 and BLM helicases in chemotherapeutics-induced glioma cell responses

**DOI:** 10.1186/s12885-025-14932-0

**Published:** 2025-09-29

**Authors:** Kamil Wojnicki, Bartosz Wojtas, Iwona A. Ciechomska, Beata Kaza, Matthew Guille, Waldemar Priebe, Bozena Kaminska

**Affiliations:** 1 Laboratory of Molecular Neurobiology, Nencki Institute of Experimental Biology of the Polish Academy of Sciences, Warsaw, Poland; 2 Children’s Research Center, University Children’s Hospital Zurich, University of Zurich, Zurich, Switzerland; 3 Laboratory of Sequencing, Nencki Institute of Experimental Biology of the Polish Academy of Sciences, Warsaw, Poland; 4 School of Biological Sciences, European Xenopus Resource, University of Portsmouth, Portsmouth, PO1 2UP UK; 5 The University of Texas MD Anderson Cancer Center, Houston, TX USA

**Keywords:** RecQ helicases, Glioblastoma, PARP inhibitors, cellular senescence, polyploidy

## Abstract

**Objectives:**

Human RECQL4 and BLM helicases participate in all DNA dependent processes, including replication stress, DNA damage repair. Both helicases are overexpressed in glioblastoma (GBM), a lethal primary brain tumour, characterised by resistance to radio- and chemotherapy. BLM-depleted glioma cells exhibit senescence-associated or polypoid phenotype when exposed to temozolomide (TMZ) and olaparib (OLA), a PARP inhibitor. This study aims to investigate how RECQL4 depletion influences the response of malignant gliomas to chemotherapeutics.

**Methods:**

We investigated the effect of RECQL4 depletion in glioma cells on cell growth, apoptosis, senescence and polyploidy in the response to combined TMZ and OLA treatment. We compared transcriptomes of RECQL4- and BLM-depleted LN18 and LN229 glioma cells. Drug-induced cytotoxicity, senescence-associated phenotypes, cell cycle alterations, and polyploidy were assessed using the MTT metabolic assay, β-galactosidase activity assay, and propidium iodide staining.

**Results:**

RECQL4 depletion modestly affected basal glioma cell viability and proliferation, similarly to knock out of the BLM protein. Deletion of RECQL4 in glioma cells (RQ4 KO) induced profound transcriptomic alterations, dissimilar to BLM depletion. RECQL4-depleted glioma cells treated with TMZ and OLA exhibited reduced viability and increased levels of apoptosis markers. The treatment induced cell cycle arrest, however, RQ4 KO cells did not show signs of senescence phenotype or polyploidisation, when compared to BLM KO glioma cells. Interestingly, both RQ4 KO and BLM KO cells were more resistant to WP744, a doxorubicin derivative, when compared to WT LN229 glioma cells.

**Conclusion:**

Our results highlight the distinct roles of RecQ helicases in a response to chemotherapeutics and support a rationale for targeting RECQL4 as a therapeutic strategy in glioblastoma.

**Supplementary Information:**

The online version contains supplementary material available at 10.1186/s12885-025-14932-0.

## Introduction

RECQL4 helicase is a member of the highly conserved RecQ family of DNA helicases [1], involved in the ATP-dependent DNA unwinding and the maintenance of genome integrity [2–4]. RecQ helicases mediate genome replication, DNA repair, recombination, transcription, and translation. Germline defects in *RECQL4* and other genes from the family: *RECQL1*,* BLM*, *WRN* result in syndromes associated with premature aging and predisposition to cancer [5–7]. Mice harbouring mutations or targeted deletions of *Recql4* exhibit limited survival. Surviving individuals displayed premature aging phenotypes. The somatic *Recql4* deletion in adult mice results in severe hematopoietic dysfunctions [8]. *RECQL4* knockdown in HeLa cells caused chromosome misalignment and delayed mitotic progression, independently from the RECQL4 role in DNA replication and damage repair [4].

Glioblastoma (GBM) is an aggressive, primary brain tumour, characterised by high proliferation, infiltrative growth, genomic instability, aberrant angiogenesis and resistance to standard therapies involving surgical resection, radio- and chemotherapy with temozolomide (TMZ) [9]. The prognosis remains unfavourable, with a median survival of 14,6 months post diagnosis [10]. The poly (ADP-Ribose) polymerase inhibitors (PARPi) interfere with the DNA repair pathway and showed the potential to enhance TMZ toxicity. Clinical trials of PARPi with TMZ (the OPARATIC trial) have been launched in GMB patients [11–13]. We found markedly upregulated RECQL4 and BLM at mRNA and protein levels in GBMs, while their protein levels were low in lower grade gliomas. Notably, high expression of *RECQL4* and *BLM* corresponded to poor survival of GBM patients. Knockout of RECQL4 in LN18 and LN229 human glioma cells using the CRISPR/Cas9 editing, resulted in profound changes in the transcriptome, reduced glioma sphere formation capabilities and augmented vulnerability to TMZ [14]. Deletion of BLM in aforementioned glioma cells induced significant transcriptomic alterations, reduced cell proliferation, and altered responses to TMZ with OLA. BLM-depleted LN229 cells (partially functional p53^mut^) were more susceptible to TMZ in contrast to BLM KO LN18 cells (non-functional p53^mut^). Interestingly, BLM knockout in LN18 and LN229 cells induced resistance to the combined TMZ and OLA treatment, resulting in polyploidy or cellular senescence, respectively [15] suggesting new facets of BLM involvement in DNA repair or an apoptotic cell death.

This study evaluated the response of RECQL4-depleted cells to chemotherapeutic agents, including the combination of TMZ and OLA, as well as WP744, a doxorubicin derivative, aiming to elucidate the specific roles of the RECQL4 and BLM helicases in the therapeutic response of GBM cells. Comparison of the transcriptional profiles of RECQL4- and BLM-depleted cells revealed significant differences in genes involved in cell cycle regulation and receptor tyrosine kinase signalling pathways. RECQL4 depletion abolished the cytotoxic effect of the combined TMZ and OLA treatment in LN18 and LN229 cells. However, in contrast to BLM KO cells, RECQL4-deficient cells did not exhibit polyploidisation or features of cellular senescence. Moreover, the cytotoxic response of RECQL4 KO glioma cells to TMZ and OLA remained comparable to that of control cells. In contrast, both RECQL4 KO and BLM KO LN229 cells showed increased resistance to WP744, a doxorubicin derivative that induces apoptosis. These findings underscore the critical role of RecQ helicases in malignant glioma biology and reveal functional divergence between RECQL4 and BLM in the context of therapeutic response.

## Materials and methods

### Cell cultures and treatments

LN18 and LN229 glioma cell lines were purchased from the American Type Culture Collection (ATCC, Manassas, VA, USA) and cultured in DMEM medium (Dulbecco’s modified Eagle medium, Thermo Fisher Scientific). LN18 and LN229 RECQL4 and BLM knockout cells (LN18 RQ4 KO, LN229 RQ4 KO; LN18 BLM KO, LN229 BLM KO) were generated using CRISPR/Cas9 genome editing and characterised as described [14, 15]. Cells were maintained in DMEM culture media supplemented with 10% FBS (Gibco), antibiotics (100 U/mL penicillin, 100 µg/mL streptomycin) in a humidified atmosphere of CO_2_/air (5%/95%) at 37 °C.

Cells were treated with 500 µM temozolomide (TMZ, Sigma-Aldrich, vehicle: DMSO) alone or in combination with 1 µM olaparib (OLA, MedChemExpress, vehicle: DMSO). WP744, a doxorubicin derivative and topoisomerase II inhibitor, was synthesized and kindly provided by Dr. Waldemar Priebe [16]. The compound was dissolved in H_2_O prior to use.

### RNA isolation, library preparation, sequencing and bioinformatic analysis

RNAseq data were collected and analysed as previously described [14, 15]. Briefly, total RNA was extracted from glioma cells using RNeasy Mini kit (Qiagen, Germany) and purified on Rneasy columns. Next, RNA was used to synthesize cDNA using SuperScript™ III Reverse Transcriptase (Invitrogen). Quantity and quality of RNA was estimated using Agilent Bioanalyzer.

Quality and integrity of RNA was assessed with Agilent 2100 Bioanalyzer using a RNA 6000 NanoKit (Agilent Biotechnologies). PolyA enriched RNA libraries were prepared using the KAPA Stranded mRNA Sample Preparation Kit (Kapa Biosystems). Transcriptomic data were analysed as follows: fastq files were aligned to hg38 human reference genome with STAR program [17], and reads were counted to genes using feature Counts algorithm SUBREAD package [18]. Gene counts were normalized with the FPKM method, and differential analysis was performed using the DESeq2 [19]. Genes were considered to be differentially expressed (DE) with FDR corrected p-value < 0.05. Kyoto Encyclopedia of Genes and Genomes (KEGG) pathway analyses were performed using R package clusterProfiler [20] to annotate the functions of differentially expressed mRNAs. Differential expression analysis was performed between RQ4 WT, BLM WT versus the respective KO groups, and then DEGs were compared between RQ4 KO and BLM KO in respective cell lines.

### Cell viability and proliferation

Cells were seeded at a density of 4-5 × 10^3^ cells/well and cell viability was determined using MTT or PrestoBlue™ reagent according to manufacturer’s recommendation. On the day of cell harvesting, the medium was replaced with fresh containing MTT or PrestoBlue™ reagent at 1:10 ratio. Following 1 h incubation, the absorbance at wavelength of 570 nm and 620 nm was measured using a scanning multi-well spectrophotometer. Cell viability measurements were normalized to the protein content in a given sample. Cell proliferation was determined using ELISA BrdU kit (Roche Diagnostics GmbH) according to the manufacturer’s protocol. For BrdU assays, cells were seeded at a density of 4 × 10^3^ cells/well and BrdU reagent was added for 2 h.

### Western blot analysis

The total protein extracts collected as described [21] were resolved on 8% or 10% SDS-PAGE gels (Eurogentec) and transferred onto nitrocellulose membranes (Bio-Rad). The proteins were probed with primary antibodies listed in the Table S1. Immunocomplexes were detected using SuperSignal West Pico^PLUS^ Chemiluminescent Substrate (Thermofisher Scientific) and visualised by ChemiDoc Imaging System (Bio-Rad Laboratories). Anti-GAPDH antibody was used as control for equal protein loading. Densitometric analyses were performed using Image Lab ver. 5.2 software (Bio-Rad Laboratories). Full length, original and unprocessed immunoblots, are presented in the Fig. S2. Due to big differences in the size of detected proteins a membrane after transfer was typically cut into 2 or 3 parts and each one of them was processed with different antibody.

### Cell cycle and cellular granularity analyses

For cell cycle and cellular granularity analysis, cells were seeded at a density of 2 × 10^5^ cells/well. Cell cycle analysis was performed by flow cytometry using BD Pharmingen PI/RNase Staining Buffer (BD Biosciences). Briefly, cells were collected by trypsinisation, fixed in 70% ethanol and stained in PI buffer (500 µL/1 × 10^6^ cells). DNA content analyses were performed using FACScalibur flow cytometer (BD Biosciences) and the BD CellQuest Pro 6.0 software (BD Biosciences). At least 10,000 events were analysed for each sample.

### F-actin staining with Rhodamine phalloidin

Cells were seeded onto glass coverslip at a density of 3 × 10^4^ cells. Following 24 h incubation, cells were treated with TMZ and OLA for 48 h. Next, cells were washed with PBS, fixed with 2% paraformaldehyde in PBS and permeabilised with 0.1% triton X-100. Rhodamine-Phalloidin (1:1000 in PBS) was used to stain F-actin for 25 min at room temperature and then co-stained with DAPI (1 µg/mL) to visualize cell nuclei. Images were acquired with fluorescent microscopy.

### Quantification of senescence-associated β-galactosidase-positive cells

The activity of senescence-associated β-galactosidase (SA-β-gal) was detected, as described [15]. Briefly, cells were seeded at density 2 × 10^4^ cells and after the treatment fixed with 2% formaldehyde and 0.2% glutaraldehyde in PBS, washed, and incubated overnight at 37 °C in the solution containing 1 mg/mL 5-bromo-4-chloro-3-indolyl-β-D-galactopyranoside, 5 mM potassium ferrocyanide, 5 mM potassium ferricyanide, 150 mM NaCl, 2 mM MgCl_2_, and 0.1 M phosphate buffer, pH 6.0. Cells were visualised under a Nikon Eclipse 50i microscope (Minato) and counted. Percentages of SA-β-gal-positive cells were calculated.

### Statistical analysis

All biological experiments were performed on independent 3–4 cell passages. Results are expressed as means ± standard deviation (SD). P values were calculated using chi-square test, two-tailed t-test, one-way or two-way ANOVA followed by appropriate post-hoc test and ANOVA contrast. Most analyses have been done using GraphPad Prism v6 (GraphPad Software). Moreover, we calculated the odds ratio (OR), and effect size (Hedge’s ‘g’) between the groups. A commonly used interpretation for effect size is as follow: small (0.2), medium (0.5) and large (0.8), however these values are arbitrary and should not be considered rigidly [22, 23].

## Results

### RECQL4 or BLM depletion in glioma cell lines altered marginally cell viability and cell proliferation

**Fig. 1:**
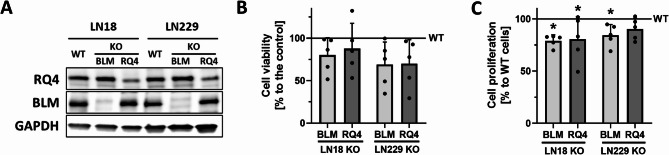
RECQL4 or BLM depletion in glioma cells altered marginally cell viability and cell proliferation. **A** Representative immunoblot shows levels of RECQL4 and BLM proteins in WT and respective depleted glioma cells. **B** Cell viability determined by MTT metabolism assay of WT, RQ4 KO and BLM KO LN18 and LN229 glioma cells after 72 h post seeding (*n* = 5). **C** Cell proliferation measured by BrdU assay of WT, RQ4 KO and BLM KO of LN18 and LN229 glioma cells. Black solid line indicates values in control, WT cells. Statistical analysis was performed using Kruskal-Wallis test with Dunn’s post-hoc test between WT and KO cells, (*n* = 5 in triplicates). Bars plotted with the mean ± SD, *p < 0.05

We analysed the levels of RECQL4 and BLM proteins in LN18 and LN229 glioma cells depleted of respective proteins. Development of those cell lines has been previously reported and validated [14, 15]. Notably, the RECQL4 depletion was not complete, whereas knock out of BLM was efficient (Fig. 1A, S1A). RECQL4 or BLM depletion in glioma cells moderately affected cells viability, however LN229 cells displayed minimally increased sensitivity to helicases knock out (Fig. 1B). Moreover, RECQL4 and BLM KO reduced cell proliferation in glioma cells in a range of 10–20% (Fig. 1C).

### Transcriptomic profiling of RECQL4 and BLM depleted LN18 and LN229 cells highlights extracellular matrix remodelling as a top effected pathway

**Fig. 2:**
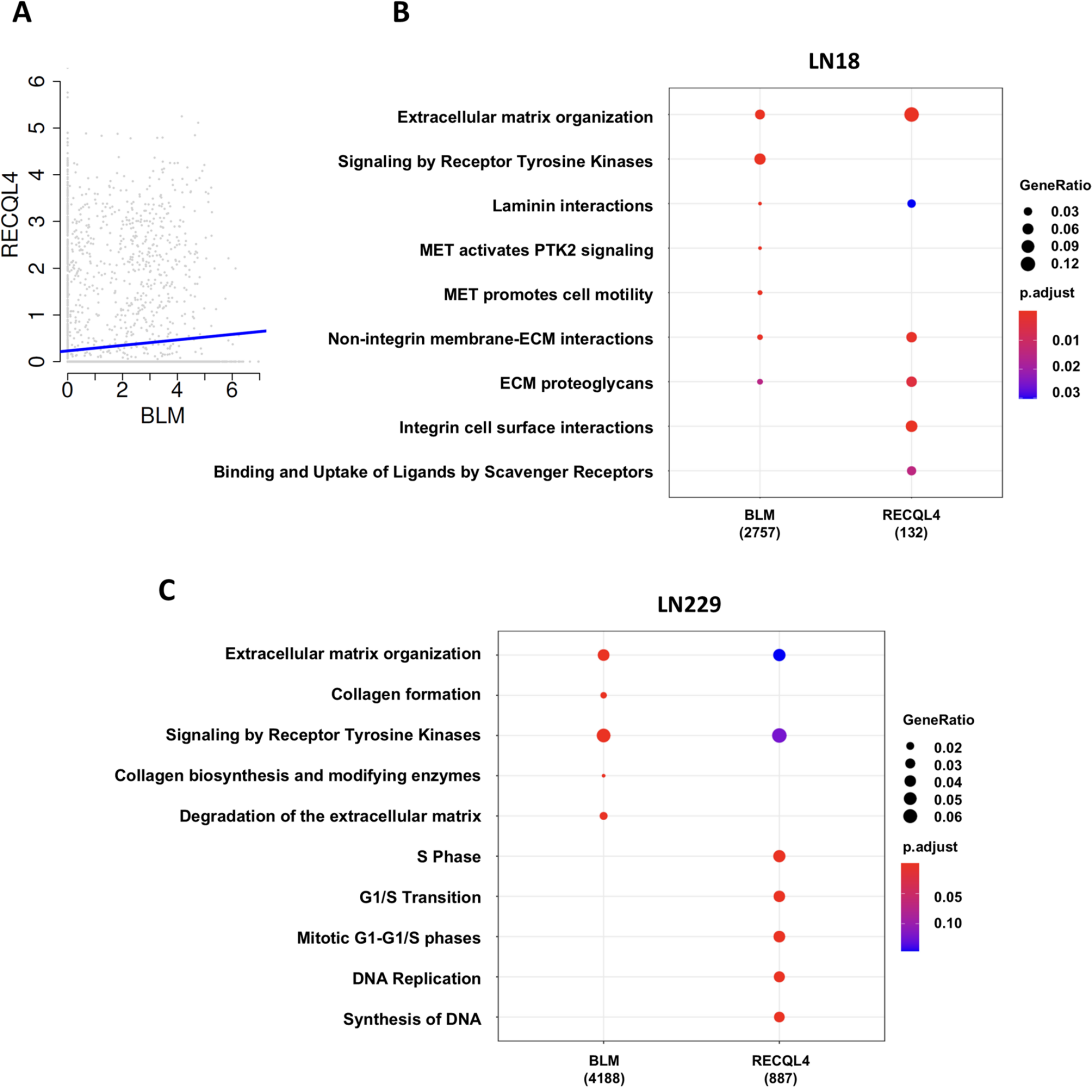
Comparison of GO functional pathways resulting from transcriptomic changes in RECQL4 and BLM KO glioma cells. **A** RECQL4 and BLM expression in GBM cells (malignant annotation from Neftel et al. 2019 [20]), was depicted on the y/x axis, where each dot is a single cell. Positive correlation of RECQL4 and BLM genes was significant (correlation = 0.11, p-value < 2.2e-16). **B-C** KEGG analysis of differentially expressed genes (FDR corrected *p* < 0.05) displays deregulated pathways in LN18 (**B**) and LN229 BLM (**C**) KO cells when compared to respective WT controls

We used scRNSseq dataset generated from 28 tumours from Neftel et al. 2019 [20] for the analysis of RECQL4 and BLM expression in GBM cells. Malignant cell annotation was taken from [20]. We found the significant, positive correlation of *RECQL4* and *BLM* gene expression in malignant cells (correlation = 0.11, p-value < 2.2e-16, single dot represents a single cell) (Fig. 2A).

To determine similarities or differences in transcriptional networks regulated by RECQL4 and BLM helicases, we performed the comparative analysis using previously generated RNAseq datasets (the NIH GEO database the accession numbers: GSE285044 for RECQL4 and GSE214931 for BLM). We focused on significant transcriptomics changes (differentially expressed genes, DEGs) in LN18 and LN229 KO glioma cells in comparison to respective WT controls, and compared DEGs in RQ4 KO and BLM KO cells. DEGs were analysed for enriched functional categories using Kyoto Encyclopedia of Genes and Genomes (KEGG) pathway analyses.

The graph shows that depletion of either RECQL4 or BLM has different consequences in different glioma cells (Fig. 2B-C), with disturbances of cell cycle related genes noteworthy in RQ4 KO LN229 cells (Fig. 2C). Interestingly, the top affected pathways among DEGs in both RQ4 and BLM KO LN18 and LN229 cells are genes related to extracellular matrix (ECM) organization, degradation of ECM, collagen modification and synthesis which were changed in both cell lines and KO cells. (Fig. 2B, C). The pathways related to cell cycle were enriched exclusively in RQ4 KO LN229 cells (Fig. 2C). ECM organization, integrin and non-integrin membrane ECM interactions were among top affected pathways in RQ4 KO cells. These transcriptomics analyses show that ECM organization is one of the prominent and shared pathways between RQ4 and BLM KO glioma cells, while RECQL4 deficiency has a stronger impact on the cell cycle than BLM deficiency.

### RECQL4 deficiency has no impact on the responses of glioma cells to the combined, TMZ and OLA, treatment

**Fig. 3:**
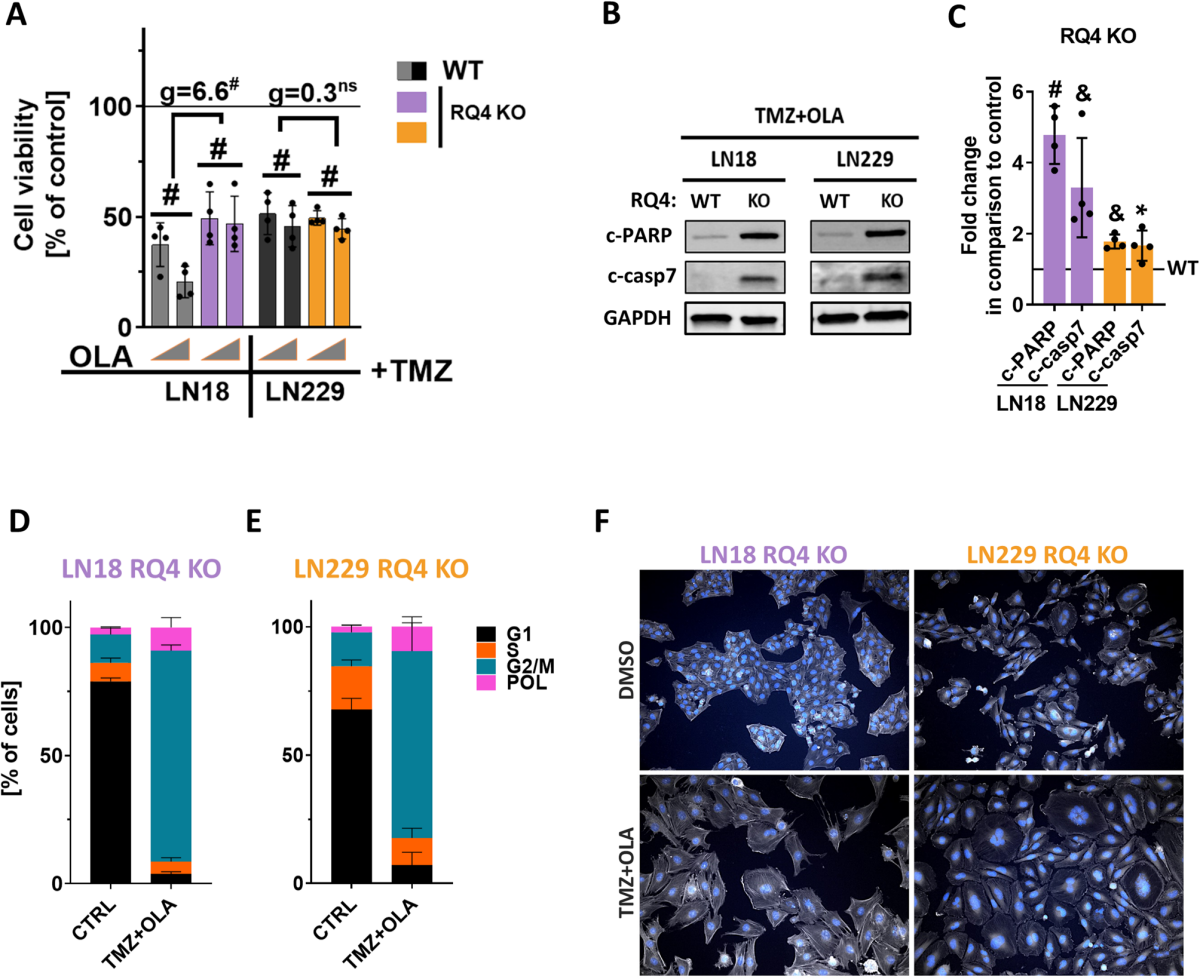
RECQL4 deficiency in glioma cells does not sensitize glioma cells to TMZ and OLA treatment. **A** Viability of WT or RQ4 KO LN18 and LN229 cells after double, TMZ and OLA, treatments, determined by MTT metabolism test 72 h after the treatments. Cell viability of control cells set as 100% is represented by a black solid line. Grey triangles represent increasing doses of OLA (1 and 5 µM) with 250 µM TMZ. Statistical analysis was performed using linear contrast ANOVA analysis (^#^*p* < 0.001), mean ± SD, *n* = 4. Hedge’s ‘g’ stands for effect size. **B** Representative immunoblots showing upregulation of cleaved, apoptotic protein (c-PARP, c-casp7) levels in RQ4 KO LN18 and LN229 cells after TMZ and OLA in comparison to WT cells. GAPDH was used as a loading control. **C** Densitometric analysis of immunoblots from 3 experiments. Statistical significance was determined by one sample t-test on logarithmic raw data *p < 0.05, ^&^*p* < 0.01, ^#^*p* < 0.001), *n* = 3, mean ± SD. **D**,** E** Percentages cells in the cell cycle phases in cultures of RQ4 KO and WT LN18 (**D**) and LN229 (**E**) cells after the TMZ and OLA treatments, determined using the propidium iodide staining and flow cytometry, *n* = 3, ≥ 10000 events/sample, mean ± SD. **F** Representative images of F-actin staining of RQ4 KO LN18 and LN229 cells treated with 500 µM TMZ and 1 µM OLA (TMZ + OLA) for 48 h; DMSO served as a control. Nuclei were visualised using DAPI staining (total magnification 200x)

Cell viability was measured using MTT assay. The combined, TMZ and OLA, treatment resulted in decreased viability in LN18 and LN229 cells, irrespectively of the RECQL4 status. Moreover, there was no differences between treated RQ4 WT (black bars) and KO (orange bars) LN229 cells (g = 0.3), whereas RQ4 KO (violet bars) LN18 cells displayed a resistance to the combined treatment when compared to RQ4 WT (grey bars) cells (g = 6.6) (Fig. 3A). Interestingly, the TMZ and OLA combination induced a strong upregulation of c-PARP, c-casp7 levels in RQ4 KO cells when compared to RQ4 WT, as evidenced by Western blotting, which indicates the induction of apoptosis, especially in LN18 cells (violet bars) (Fig. 3B, S1A, S2B-C). These findings are corroborated by the results of the densitometric analysis (Fig. 3C). RECQL4 depletion had impact on cell cycle of glioma cells following the simultaneous TMZ and OLA treatment. RQ4 KO LN18 and LN229 cells tend to be arrested in G2/M phase, when compared to control conditions (Fig. 3D, E). Percentages of cells in each cell cycle phase are given in the table (Fig. S1B). Next, we visualised F-actin organization with rhodamine-phalloidin staining. Both LN18 and LN229 treated cells underwent morphological changes such as enlargement and flattening of cell body. Some cells showed nuclei fragmentation which is indicative of undergoing apoptotic cell death.

### Cell cycle arrest in RECQL4 deficient glioma cells is not connected to cellular senescence

**Fig. 4:**
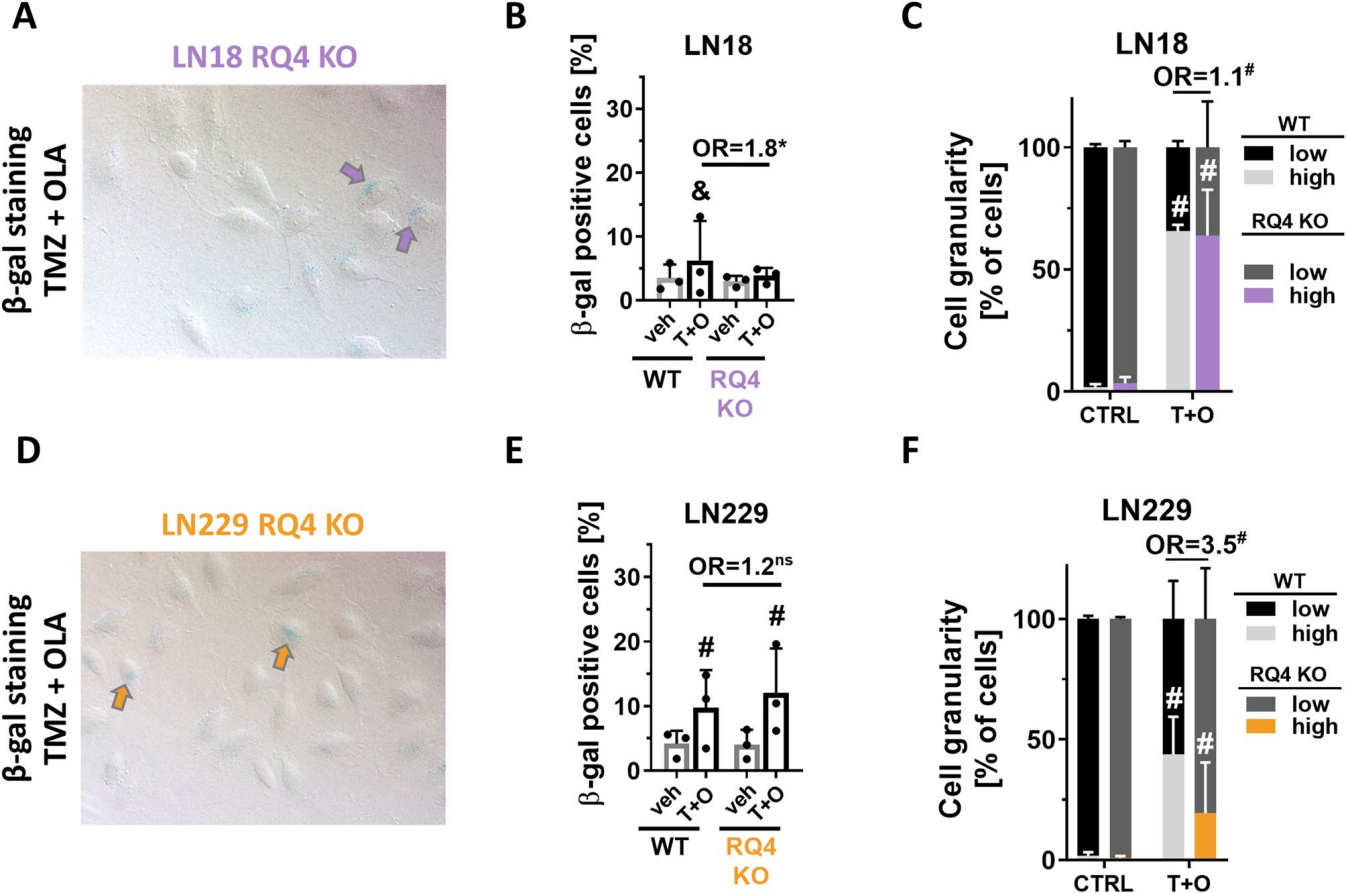
RECQL4 deficiency does not affect drug induced cellular senescence in glioma cells. **A**,** D** Representative images of β-gal staining of RQ4 KO LN18 (**A**) and LN229 (**D**) cells after the TMZ and OLA treatments. Blue colour indicates the increased activity of β-galactosidase (examples marked by arrows) (**B**, **E**) Quantification of β-gal positive cells amongst control and treated RQL4 KO (**B**) LN18 and (**E**) LN229 cells. Statistical analysis was performed using a chi-squared test in comparison of treated versus control cells (above the bars, & *p* < 0.01, # *p* < 0.001), or between the WT and RQ4 KO cells (above the lines, *p < 0.05), *n* = 3, in duplicates, ± SD. **C**,** F** Cell granularity of TMZ and OLA-treated BLM KO and WT (**C**) LN18 and (**F**) LN229 cells determined by flow cytometry. Statistical analysis was performed using chi-square test in comparison of treated to control cells (CTRL) (above the bars, # *p* < 0.001) or between the WT and RecQL4 KO cells (above the lines, # *p* < 0.001), *n* = 3, ≥ 10,000 events/sample, ± SD. OR stands for odds ratio. OR = 1.1 CI95(1.05;1.13), OR = 3.5 CI95(3.36;3.64)

Cell cycle arrest is frequently associated with a cellular senescence phenotype, characterised mainly by increased enzymatic β-galactosidase (β-gal) activity [24]. We had previously found considerable increase in BLM KO glioma cells upon TMZ with OLA treatment [15]. Interestingly, the assessment of the β-gal activity in RQ4 KO LN18 (Fig. 4A) and LN229 (Fig. 4D), and quantification of β-gal positive cells after simultaneous treatment showed slightly increased numbers of β-gal positive cells in RQ4 WT and KO LN229 cells (Fig. 4B, E). Notably, the increase of β-gal enzymatic activity was limited (cells indicated by arrows Fig. 4A, D). Accumulation of β-gal positive cells was not observed RQ4 KO LN18 cells (Fig. 4B) and was significantly lower in comparison to drug-treated LN18 WT cells (OR = 1.8). These results were in line with the flow cytometry data, showing the percentage of highly granular LN18 cells after the TMZ and OLA double treatment (Fig. 4C) with no differences between WT and RQ4 KO cells (OR = 1.1), whereas RQ4 KO LN229 cells had even lower percentage of high granular cells after TMZ and OLA treatment in comparison to WT cells (OR = 3.5) (Fig. 4F).

### Knockdown of either RECQL4 or BLM helicases in glioma cells reduces cytotoxic responses to doxorubicin derivative - WP744

**Fig. 5:**
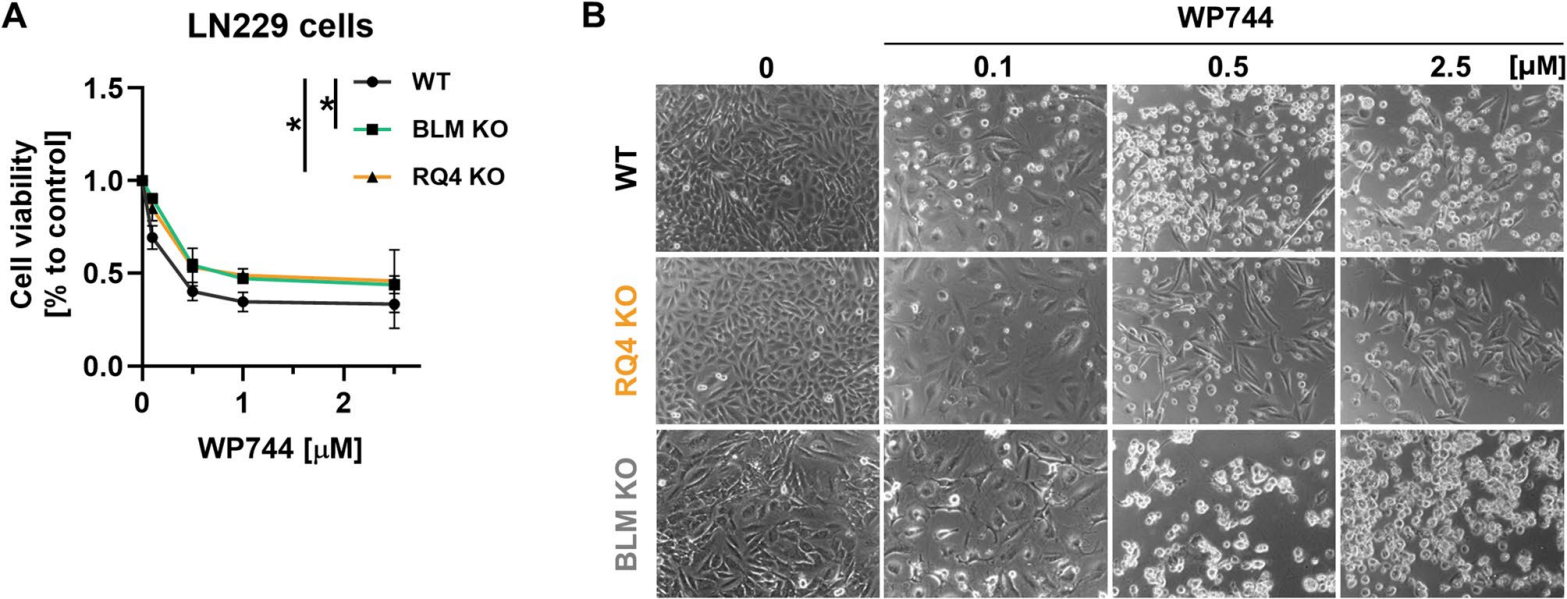
Deficiency of RECQL4 or BLM helicases in glioma cells affects WP744-induced apoptotic cell death. **A** Cell viability determined by Presto Blue of WT, RQ4 KO and BLM KO of LN229 glioma cells 24 h after WP744 treatment (*n* = 3). Statistical analysis was performed using ANOVA contrast (*p < 0.05). **B** Representative images of WT, RQ4 KO and BLM KO LN229 glioma cells after 96 h in presence of WP744. Round, detached cells represent apoptosis phenotype (total magnification 100x)

The compelling data published previously [15] and presented here, indicate the unexpected contribution of RecQL family helicases to apoptotic responses to chemotherapeutics. Therefore, we tested LN229- WT, RQ4 KO or BLM KO cells responses to WP744, the 4’-O-benzylated doxorubicin analogue, inhibiting DNA topoisomerase II and triggering apoptosis in breast cancer, acute myeloid leukemia, neuroblastoma cells and human glioma stem cells [16, 25]. Mechanistically, WP744 treatment induced apoptotic cell death, associated with cleavage of caspases-3, −9, and PARP through an increase in p53 protein levels, and the induction of the p21 cell cycle inhibitor in neuroblastoma cells [26]. As LN18 glioma cells have a mutated and nonfunctional p53, the experiments were carried out on LN229 cells. WP744 strongly impaired cell viability of WT control cells, but not RQ4 and BLM KO cells (Fig. 5A). Representative images show cell rounding and detachment in WT glioma cells treated with WP744, while RQ4 KO and BLM cells remained unchanged morphologically (Fig. 5B). The observed morphological are consistent with those induced during the apoptotic cell death.

## Discussion

RecQ helicases participate in multiple DNA-dependent processes essential for genome replication, repair, recombination and transcription. While some functions are overlapping between RecQ family members, each of RecQ helicase also exhibits distinct, non-redundant roles [2, 3, 7]. In this manuscript we are demonstrating shared and non-overlapping functions of two members of RecQ family: BLM and RECQL4 (RQ4). Due to important role in DNA maintenance, the lack of those two helicases led to decreased cell proliferation and viability of LN18 and LN229 glioma cells. This observation was reported also in other cells like primary murine osteoblasts [27], cervical cancer cells [28] or prostate cancer cells [8, 29]. Furthermore, RQ4 localises to mitochondria and its lack may lead to decreased mitochondrial activity [30] and ATP level, reflected by lower MTT assay redout.

Transcriptomic analysis revealed distinct gene expression patterns between RQ4 and BLM KO glioma cells, particularly in cell cycle–related transcripts, highlighting functional differences between these two RecQ helicases. RQ4 KO cells showed pronounced dysregulation of genes involved in mitotic progression and checkpoint control, consistent with its role in DNA replication and replication fork stability [31]. In contrast, the shared upregulation of extracellular matrix-related genes, including those involved in ECM remodelling and laminin interactions, suggests a cellular adaptation to helicase deficiency, possibly linked to altered adhesion dynamics or senescence. These findings suggest that although both helicases contribute to maintaining genome stability, they do so through different mechanisms. As a result, cells lacking RQ4 or BLM may respond differently to treatments that induce replication stress or target DNA repair pathways, therefore we introduced treatments including an alkylating agent (temozolomide, TMZ) together with PARP1 inhibitor (olaparib, OLA), and topoisomerase II inhibitor (WP744). The application of PARP inhibitors represents a promising strategy for targeting tumor cells with defects in the DNA repair systems [32]. PARPi combined with TMZ showed the promising cytotoxic effects against GBM cells and tumors in rodents, which led to clinical trials of PARPi with TMZ in GMB patients (the OPARATIC trial) [12, 13, 33]. However, elevated expression of RECQL4 in GBMs or loss of function mutations in the *RECQL4* gene [14, 34] could interfere with drug effectiveness. Therefore, we sought to determine how a lack of RECQL4 or BLM in glioma cells would affect cell responses to therapy.

Our previous study demonstrated that BLM knockout in LN18 and LN229 cells yielded cells resistant to the combined TMZ + OLA treatment, leading to polyploidy in LN18 cells and cellular senescence in LN229 cells [15] suggesting a role of BLM in drug-induced cell death. RECQL4 depletion reduced cytotoxicity of TMZ + OLA in LN18 cells, however a fraction of cells underwent apoptosis as the levels of cleaved caspase 3 and 7 were augmented in RQ4 KO cells, particularly in LN18 cells. In contrast to BLM KO cells, such treatment did not induce senescence (based on β-gal-positive cells) or polyploidisation in RQ4 KO glioma cells. TMZ with OLA induced the growth arrest of treated RQ4 cells in the G2/M phase and morphological changes (cells were enlarged and flat). To pinpoint more precisely which would be the involvement of RECQL4 and BLM helicases to cell death process, we employed WP744, a 4’-O-benzylated doxorubicin analogue, which induces the apoptotic, p53-dependent cell death in many tumor cells, including glioblastoma cells [16, 25]. WP744 effectively impaired WT human glioma cell viability, when compared to RQ4 KO or BLM KO cells. The observed changes in cell morphology such as rounding and detachment of treated WT cells are consistent with ongoing apoptotic cell death, while RQ4 KO cells were more resistant and remained morphologically unchanged. As WP744 induces the apoptotic cells death in glioma cells [26], the presented results suggest specific functions for RECQL4 and BLM helicases in chemotherapeutics- induced apoptosis.

The presented findings emphasise the important role of RecQ helicases in malignant glioma progression and functional differences between these helicases in responses to therapy. The results show the resistance of RECQL4 or BLM depleted cells to specific treatments calling for consideration of patient characteristics for mutations in genes coding for various RecQL helicases.

## Supplementary Information

Supplementary Material 1: Figure 1. (A) Additional immunoblots for the Fig. 3B showing upregulation of cleaved, apoptotic protein (c-PARP, c-casp3, c-casp7) levels in RQ4 KO LN18 cells after TMZ and OLA in comparison to WT cells. GAPDH was used as a loading control. B Cell cycle analysis for control and treated glioma cells in Fig. 3D, E. The table summarises percentages of cells in cell cycle phases.

Supplementary Material 2: Figure 2. (A-C) Original membranes for Western immunoblots for the Fig. 1A (A), 3B (B) and S1A (C).

Supplementary Material 3.

## Data Availability

No datasets were generated or analysed during the current study.
