# Supplementary figures and images for: Shared and non-overlapping functions of RECQL4 and BLM helicases in chemotherapeutics-induced glioma cell responses
Source: BMC Cancer. 2025 Sep 29;25:1434. doi: 10.1186/s12885-025-14932-0 (PMC12482529; doi:10.1186/s12885-025-14932-0)

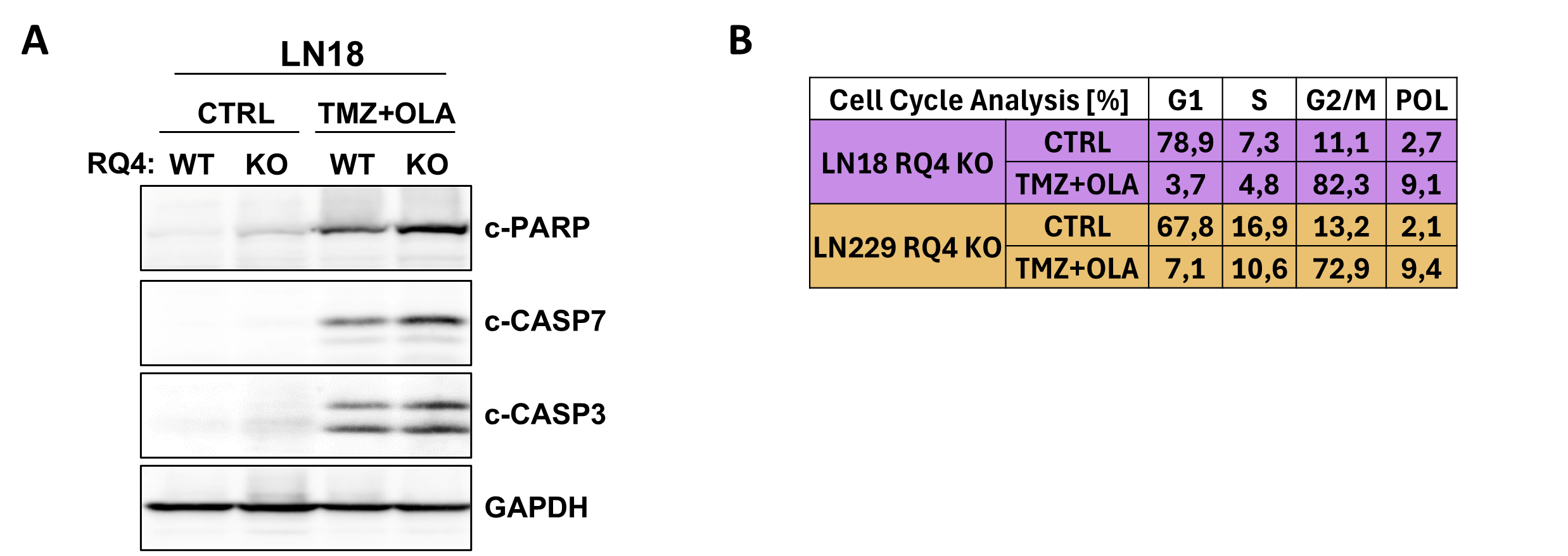

Supplement: Supplementary file 1 — Supplementary Material 1: Figure 1. (A) Additional immunoblots for the Fig. 3B showing upregulation of cleaved, apoptotic protein (c-PARP, c-casp3, c-casp7) levels in RQ4 KO LN18 cells after TMZ and OLA in comparison to WT cells. GAPDH was used as a loading control. B Cell cycle analysis for control and treated glioma cells in Fig. 3D, E. The table summarises percentages of cells in cell cycle phases. [file 12885_2025_14932_MOESM1_ESM.tif]

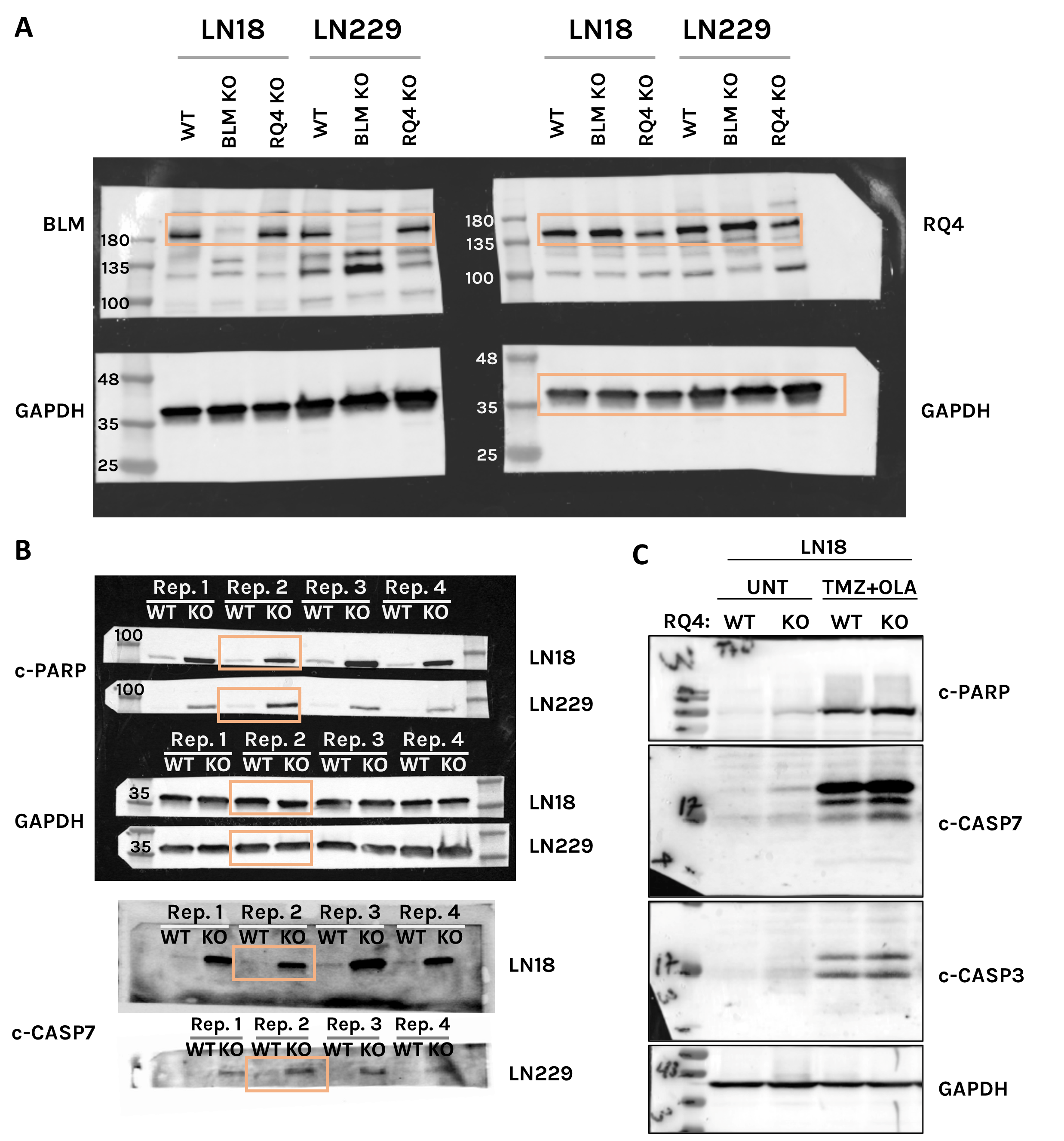

Supplement: Supplementary file 2 — Supplementary Material 2: Figure 2. (A-C) Original membranes for Western immunoblots for the Fig. 1A (A), 3B (B) and S1A (C). [file 12885_2025_14932_MOESM2_ESM.tif]
