# Supplementary material for: Shared and non-overlapping functions of RECQL4 and BLM helicases in chemotherapeutics-induced glioma cell responses
Source: BMC Cancer. 2025 Sep 29;25:1434. doi: 10.1186/s12885-025-14932-0 (PMC12482529; doi:10.1186/s12885-025-14932-0)
Supplement: Supplementary file 3 — Supplementary Material 3. [file 12885_2025_14932_MOESM3_ESM.docx]

| **Antibody** | **Clone** | **Manufacturer** | **Cat. number** | **Dilution** |
| --- | --- | --- | --- | --- |
| **Antibodies used for immunoblotting** | | | | |
| anti-BLM | - | Abcam | ab2179 | 1:1000 |
| Anti- RecQL4 | - | Novus Biologicals | 25470002 | 1:1000 |
| anti-cleaved PARP | - | Cell signaling | 9541S | 1:1000 |
| anti-cleaved Caspase 3 | - | Cell signaling | 9661S | 1:1000 |
| anti-cleaved Caspase 7 | - | Cell signaling | 9491S | 1:1000 |
| anti-GAPDH |  | Millipore | MAB374 | 1:1000 |
| horseradish peroxidase-conjugated anti-rabbit IgG | - | Vector | PI-1000 | 1:10000 |
| horseradish peroxidase-conjugated anti-mouse IgG | - | Vector | PI-2000 | 1:10000 |
| **Other reagents** | | | | |
| **Reagent** | | | **Source** | |
| Temozolomide | | | Sigma-Aldrich | |
| Olaparib | | | MedChemExpress | |
| WP744 | | | Kind gift from Waldemar Priebe, PhD | |
| Rhodamine-Phalloidin (# P1951) | | | Sigma-Aldrich | |
| Cell Proliferation ELISA, BrdU colorimetric kit (# 11647229001) | | | Roche | |
| BD Pharmingen PI/RNase Staining Buffer (# 550825 lot:7227966) | | | BD Biosciences | |
| **Software** | | | | |
| GraphPad Prism 6.07 | | | | |
| FlowJo 10 | | | | |
| BD CellQuest Pro 6.0 | | | | |

**Table S1 Antibodies, reagents and software**

Specifications, catalogue numbers and dilutions of reagents used for experiments.
